# Hypsarhythmia or Hypsarrhythmia?

**DOI:** 10.15844/pedneurbriefs-29-8-7

**Published:** 2015-08

**Authors:** John J. Millichap, J. Gordon Millichap

**Affiliations:** 1 Division of Neurology, Ann & Robert H. Lurie Children's Hospital of Chicago, Chicago, IL; 2 Departments of Pediatrics and Neurology, Northwestern University Feinberg School of Medicine, Chicago, IL

**Keywords:** EEG, Epilepsy, Infantile Spasms

## Abstract

Hypsarhythmia was originally spelled with one ‘r’ by Drs Frederick and Erna Gibbs when they coined the term in 1952.

Hypsarhythmia was originally spelled with one ‘r’ by Drs Frederick and Erna Gibbs when they coined the term in 1952 [1]. They wished to emphasize that “the term applied to a specific type of electroencephalographic abnormality”, and preferred the specific one ‘r’ spelling to avoid confusion with a literal translation of the two ‘r’ Greek root, “mountainous arrhythmia” [2, 3]. The one ‘r’ spelling was the rule in the 1950s-60s [4]. The two ‘r’ spelling eventually became convention in the literature by the mid-1970s (Figure 1).

**Figure 1:**
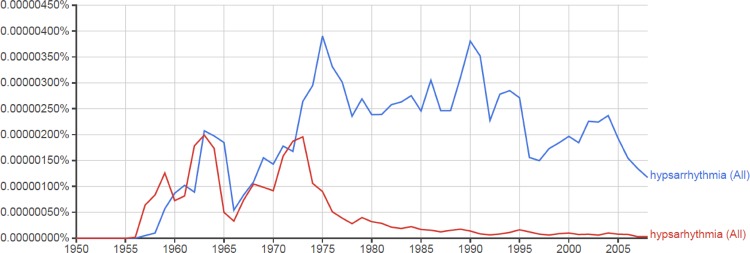
Google Ngram Viewer: Book text search for case-insensitive ‘Hypsarrhythmia’ (blue line) and ‘Hypsarhythmia’ (red line) from 1950-2008 in English. [Cited August 31 2015.] Available from: https://goo.gl/sJI1uG

Although the Editor of *Pediatric Neurology Briefs* prefers the single ‘r’ spelling in deference to Dr Gibbs, the intended meaning is accepted for both spellings today.
